# Strategies to Screen Anti-AQP4 Antibodies from Yeast Surface Display Libraries

**DOI:** 10.3390/antib11020039

**Published:** 2022-06-05

**Authors:** Aric Huang, Wei Jin, Ahmed S. Fahad, Brooklyn K. Mussman, Grazia Paola Nicchia, Bharat Madan, Matheus Oliveira de Souza, J. Daniel Griffin, Jeffrey L. Bennett, Antonio Frigeri, Cory J. Berkland, Brandon J. DeKosky

**Affiliations:** 1 Department of Pharmaceutical Chemistry, The University of Kansas, Lawrence, KS 66044, USA; huanga@ku.edu (A.H.); jinwei1031@gmail.com (W.J.); afahad@mgh.harvard.edu (A.S.F.); fbharat@mgh.harvard.edu (B.M.); matheus@ku.edu (M.O.d.S.); berkland@ku.edu (C.J.B.); 2 Department of Chemical Engineering, The University of Kansas, Lawrence, KS 66044, USA; mussman.brooklyn@gmail.com; 3 Department of Biosciences, Biotechnologies and Biopharmaceutics, University of Bari Aldo Moro, 70121 Bari, Italy; graziapaola.nicchia@uniba.it; 4 Bioengineering Graduate Program, The University of Kansas, Lawrence, KS 66045, USA; dannyg.phd@gmail.com; 5 Departments of Neurology and Ophthalmology, Programs in Neuroscience and Immunology, University of Colorado at Anschutz Medical Campus, Aurora, CO 80045, USA; jeffrey.bennett@cuanschutz.edu; 6 Department of Basic Medical Sciences, Neurosciences and Sense Organs, School of Medicine, University of Bari Aldo Moro, 70121 Bari, Italy; antonio.frigeri@uniba.it; 7 Department of Chemical Engineering, Massachusetts Institute of Technology, Cambridge, MA 02139, USA; 8 The Ragon Institute of MGH, MIT, and Harvard, Cambridge, MA 02139, USA

**Keywords:** yeast display, antibody discovery, yeast library screening, biopanning, aquaporin-4

## Abstract

A rapid and effective method to identify disease-specific antibodies from clinical patients is important for understanding autoimmune diseases and for the development of effective disease therapies. In neuromyelitis optica (NMO), the identification of antibodies targeting the aquaporin-4 (AQP4) membrane protein traditionally involves the labor-intensive and time-consuming process of single B-cell sorting, followed by antibody cloning, expression, purification, and analysis for anti-AQP4 activity. To accelerate patient-specific antibody discovery, we compared two unique approaches for screening anti-AQP4 antibodies from yeast antibody surface display libraries. Our first approach, cell-based biopanning, has strong advantages for its cell-based display of native membrane-bound AQP4 antigens and is inexpensive and simple to perform. Our second approach, FACS screening using solubilized AQP4 antigens, permits real-time population analysis and precision sorting for specific antibody binding parameters. We found that both cell-based biopanning and FACS screening were effective for the enrichment of AQP4-binding clones. These screening techniques will enable library-scale functional interrogation of large natively paired antibody libraries for comprehensive analysis of anti-AQP4 antibodies in clinical samples and for robust therapeutic discovery campaigns.

## 1. Introduction

Around 60% of approved therapeutic drugs target membrane proteins [1,2], which are important drug targets due to their involvement in a variety of cellular functions such as facilitating molecular transport across the plasma membrane, cell signaling, and catalyzing reactions [3]. However, antibody discovery against transmembrane proteins is challenging because it is often difficult to purify and solubilize membrane proteins in their native form [4]. For some proteins, including aquaporin-4 (AQP4), much of the protein is embedded in the membrane, and the extracellular domains may be small and inaccessible, limiting the extracellular epitopes that can be targeted by antibodies and preventing facile solubilization of the comparatively short peptide ectodomains [4]. Moreover, for many autoimmune diseases, it is unclear what surface receptor can be used because multiple hydrophobic and surface proteins are implicated in the disease [5].

In neuromyelitis optica (NMO), the patient serum contains autoantibodies (called NMO-IgGs) that mediate central nervous system injury. Sequencing and characterizing autoantibodies from NMO patient samples traditionally involved the single-cell sorting of B cells, followed by cloning of antibody genes for subsequent analysis [6]. However, maintaining large numbers of B-cell monoclonal cultures is labor-intensive and limits the screening to a small fraction of the B-cell repertoire. Although the majority of NMO patients have autoantibodies against AQP4 membrane protein (i.e., anti-AQP4 IgGs), AQP4 also has different formats, which may be important for disease, and the difficulty of co-staining for all these different variants makes antigen selection for primary B-cell sorts even more difficult.

The AQP4 monomer has two major isoforms that differ in the intracellular N-terminal domain. A full-length isoform M1 (323 amino acids, 34.8 kDa) is produced from translation initiation at Met1, and a shorter isoform M23 (301 amino acids, 32.3 kDa) is produced from translation initiation at Met23 [7,8]. The M1 and M23 isoforms form heterotetramers in the cell membrane, and those heterotetramers further cluster and assemble into larger structures called orthogonal arrays of particles (OAPs) [7,8]. Interestingly, the M23-AQP4 isoform promotes the formation of OAPs through intermolecular N-terminus interaction between M23-AQP4 tetramers, while the M1-AQP4 isoform impairs OAP formation [9]. Recently, a new AQP4 isoform (AQP4ex) was identified [10,11], which resulted from the translation of AQP4 mRNA beyond the canonical stop codon, generating a C-terminus extension of 29 amino acids.

The discovery of antibodies targeting AQP4 and other membrane protein targets has remained a challenge. Immortalized cell surface display systems are commonly used for antibody discovery and could be used to screen antibodies against the many relevant antigen variants for autoimmune diseases, including for membrane targets [12,13]. The surface display allows expressed peptides or antibody fragments to be anchored and displayed on the cellular surfaces of the cell, and cell selection techniques can identify and isolate the variants that display favorable binding to the desired target. The process of antibody library screening can be accomplished through several methods, including cell-based biopanning or fluorescence-activated cell sorting (FACS) [12,14].

**Figure 1:**
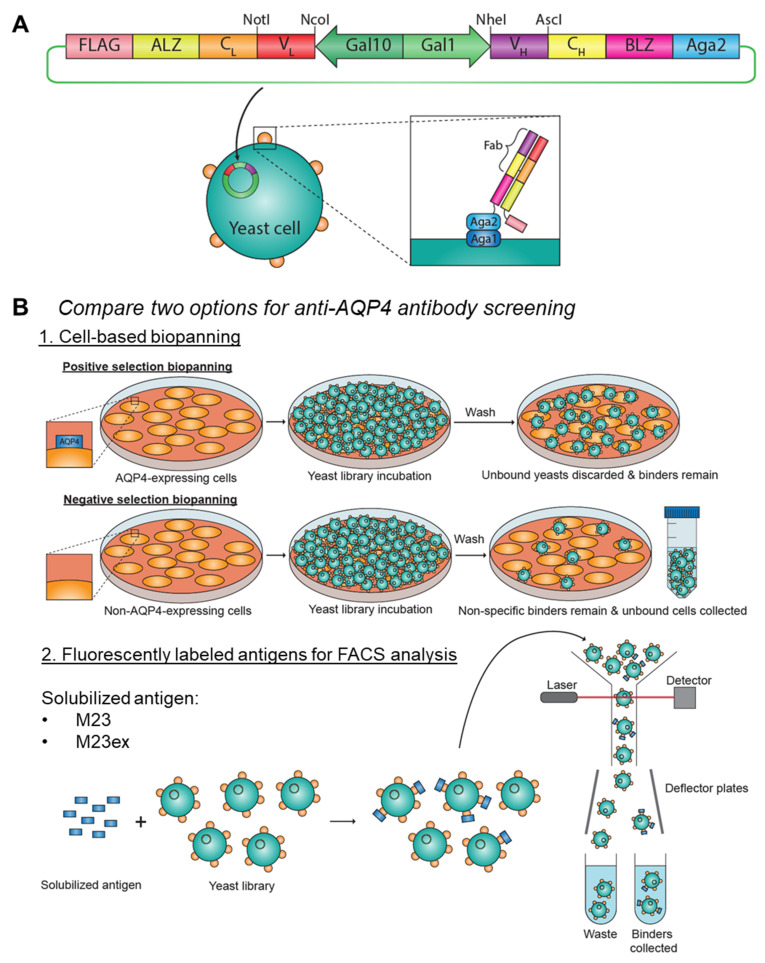
Yeast surface display and screening strategies. (**A**) Overview of the yeast surface display system. Fab expression vector contains a galactose-inducible bidirectional promoter Gal1/Gal10 for the transcription of the variable and constant heavy chains (V_H_ and C_H_), and variable and constant light (V_L_ and C_L_) chains; as well as the acidic and basic leucine-zipper (ALZ and BLZ) dimerization domains, and the FLAG protein tag. The NheI and AscI restriction sites and NotI and AscI restriction sites allow the insertion of the desired V_H_ and V_L_ sequences, respectively. Transfection of the vector allows the antibody-binding fragment (Fab) to be displayed on the surface of the yeast cell. (**B**) We compare two antibody screening strategies: (1) Cell-based biopanning strategy: For positive selection biopanning, well plates are coats with AQP4-expressing mammalian cells. After subsequent incubation with the yeast library, AQP4 binders remain on the plate. For negative selection biopanning, well plates are coated with wildtype mammalian cells that do not express AQP4. After subsequent incubation with the yeast library, non-specific binders remain on the plate and the unbound yeast cells are collected. (2) FACS screening strategy: Detergent-solubilized AQP4 membrane protein (M23 or M23ex AQP4) contain a 6xHis tag for fluorescent labeling. The solubilized AQP4 antigen are mixed with the yeast library, and the resulting mixture is sorted using FACS. Non-binding yeast clones are discarded into the waste and the binders are collected, which can be used for further enrichment or hits analysis.

To establish more effective technologies for discovering antibodies against hydrophobic surface proteins using the AQP4 antigen as an experimental model, we explored several platform approaches to identify the most effective technique to screen antibody libraries. We applied a synthetically generated yeast display library and performed cell-based biopanning and FACS library screening in parallel, combined with next-generation sequencing (NGS), to evaluate and identify successful approaches to identify AQP4-binding antibody clones. Control antibody sequences were transformed into yeast cells, generating the yeast display library where the antibody fragment is displayed on the extracellular surface of the transfected yeast cells (Figure 1A). The yeast library was then screened (via biopanning or FACS screening) for specificity to an antigen by incubating the library with the antigen (Figure 1B). The screening strategy is outlined in Figure S1. Both cell-based biopanning (using AQP4-expressing mammalian cells) and FACS screening (using solubilized AQP4 antigen) were effective at enriching AQP4-binding clones, and the screening parameters established in this study will help us to effectively screen large antibody libraries, which will help us better understand the antibody response in NMO and other autoimmune patients.

## 2. Materials and Methods

### 2.1. Cell Culture and Media

The yeast strain AWY101 (*MATα* *AGA1::GAL1-AGA1::URA3 PDI1::GAPDH-PDI1::LEU2 ura3-52 trp1 leu2**Δ**1 his3**Δ**200 pep4::HIS3 prb1**Δ**1.6R can1 GAL*) (kind gift from Eric Shusta, University of Wisconsin-Madison) was used for library construction and screening. Yeast cells were maintained in YPD medium (Sigma Y1375, Saint Louis, MO, USA); after plasmid transformation, yeast cells were maintained in SDCAA medium (Teknova 2S0540, Hollister, CA, USA). Fab surface display was induced using SGDCAA medium (SGCAA medium with galactose (Teknova 2S0542) supplemented with 2 g/L dextrose) at OD_600_ = 0.15 with incubation at 20 °C, 225 rpm for 36 h. For control, wildtype AWY101 yeast cells were supplemented with 10 μg/mL of tryptophan when in SDCAA or SGDCAA medium. In order to discourage bacterial growth during and after sorting, yeast cells were collected into and maintained in low pH SDCAA medium (20 g/L dextrose, 6.7 g/L yeast nitrogen base, 5 g/L casamino acids, citrate buffer (pH 4.5, 10.4 g/L sodium citrate and 7.4 g/L citric acid monohydrate) supplemented with penicillin–streptomycin.

U87MG cells expressing human AQP4-M23 were maintained in MEM α with nucleoside (Gibco 12571, Life Technologies Corporation, Grand Island, NY, USA) supplemented with 10% FBS, sodium pyruvate, non-essential amino acids, and 500 μg/mL G418 (Gibco 10131) for M23-expressing U87MG cells or penicillin–streptomycin for wildtype U87MG cells. Cells were maintained at 37.0 °C at 5.0% CO_2_.

Unmodified HEK293 cells and HEK293 cells expressing human AQP4-M23 or M23ex with C-terminal mCherry tag were maintained in high glucose DMEM (Gibco 11965) supplemented with 10% FBS and penicillin–streptomycin. HEK293 cells expressing M23 tagged with mCherry were supplemented with 250 μg/mL G418, while HEK293 cells that were expressing M23ex tagged with mCherry were supplemented with 4 μg/mL puromycin. Cells were maintained at 37.0 °C at 5.0% CO_2_.

### 2.2. Antigens and Antibodies

Soluble antigens were purified from Sf9 cells (i.e., AQP4-M23 and AQP4-M23ex with 6xHis tag) in 2% n-Dodecyl β-D-maltoside (DDM), 10% glycerol, 250 mM imidazole pH 7.4, 300 mM NaCl, and protease inhibitor cocktail [15]. Samples were aliquoted and stored at −80 °C.

Recombinant antibodies 2B4 and IC05-2-2 were used as negative controls (i.e., non-AQP4 antibodies). 2B4 is against measles virus nucleocapsid protein [16]; while IC05-2-2 was derived from a meningitis patient and did not bind to AQP4 or Tc cells [17]. 10-1-121, 10-1-153, 7-5-186, and 7-5-53 are anti-AQP4 antibodies with K_d_ values of 2563, 195, 15.2, and 14.8 nM, respectively [18].

### 2.3. Cell Staining

Mammalian cells were seeded at 2 × 10^4^ cells/well in 24-well plates (Corning 3524, Kennebunk, ME, USA) and incubated at 37.0 °C with 5.0% CO_2_ for 2 days. Cells were washed with DPBS containing calcium and magnesium (Gibco 14040) supplemented with 0.1% BSA (VWR 0332, Solon, OH, USA) and fixed for 15 min with 10% neutral buffered formalin. After 3 washes, human recombinant antibodies at 22.5 µg/mL (150 nM) or indicated concentrations were incubated for 2 h or overnight at 4 °C. If applicable, cells were washed and incubated with 4 µg/mL of rabbit anti-AQP4 antibodies (Proteintech 16473-1-AP, Chicago, IL, USA) in 0.1% triton-X for 1 h. After washing, cells were incubated with appropriate 1:500 diluted secondary antibodies: Alexa Fluor™ 488 goat anti-human IgG (Invitrogen A11013, Eugene, OR, USA), Alexa Fluor™ 568 goat anti-rabbit IgG (Invitrogen A11011), or Alexa Fluor™ 647 goat anti-rabbit IgG (Invitrogen A21244) for 1 h. After washing, cells were counterstained with 0.5 µg/mL of DAPI (Invitrogen D1306) or 5 µg/mL of Hoechst 33342 (Invitrogen H3570) for 15 min. Fluorescent images were acquired on an Olympus IX-81 inverted epifluorescence microscope (Melville, NY, USA) and processed on SlideBook 6 (Denver, CO, USA) or on an EVOS^®^ FL Auto Imaging System (Eugene, OR, USA) and processed on ImageJ.

### 2.4. Yeast Library Construction

For plasmid construction, DNA sequences encoding for antibodies 2B4, IC05-2-2, 10-1-121, 10-1-153, 7-5-186, and 7-5-53 were analyzed to determine their V_H_ and V_L_ sequences. From the sequences, V_H_ inserts were designed with NheI and AscI restriction sites, and V_L_ inserts were designed with NotI and NcoI restriction sites. Inserts were subcloned into a pCT-VHVL-K1 yeast display vector backbone, which has been previously reported [19]. Briefly, the pCT-VHVL-K1 and inserts were digested with the corresponding restriction enzymes, and the digested products were purified by agarose gel extraction. Digested vectors and inserts were ligated together with T4 ligase (NEB M0202, Ipswich, MA, USA), and the ligated product was transformed into chemically competent *E. coli* cells (NEB C3040). *E. coli* cells were spread onto LB agar plates containing ampicillin for selection. Single colonies were then picked and expanded overnight in LB broth containing ampicillin, and plasmids were isolated from cells using the ZymoPURE Plasmid Miniprep Kit (Zymo Research D4211, Irvine, CA, USA). The isolated plasmids were sequenced to confirm that the desired plasmids were obtained.

Monoclonal yeast was obtained by transforming the plasmids into AWY101 yeast using the Frozen-EZ Yeast Transformation II Kit (Zymo Research T2001). After yeast transformation, the monoclonal yeast was cultured in SDCAA medium and incubated at 30 °C, 225 rpm. The cells were then incubated in SGDCAA medium to induce Fab surface expression at 20 °C, 225 rpm for 36 h. In order to enrich for Fab expressing clones, yeast were stained with 1:50 diluted anti-FLAG M2-FITC antibody (Sigma F4049) in staining buffer (1x DPBS with 0.5% BSA and 2 mM EDTA, pH 7.4), and yeast that expressed Fab (i.e., stained positive for the FLAG-tag) were sorted and collected into low pH SDCAA using a BD FACSAria Fusion cytometer (San Jose, CA, USA). The gating strategy used is shown in Figure S2, which was drawn based on the wildtype AWY101 control.

In order to generate the yeast library, the V_L_+ sorted monoclonal yeast was expanded in low pH SDCAA at 30 °C, 225 rpm. Yeast cell counts were estimated using OD_600_ light transmission measurements, and monoclonal yeast were mixed so that the library contained 49% 2B4, 49% IC05-2-2, 0.5% 10-1-121, 0.5% 10-1-153, 0.5% 7-5-186, and 0.5% 7-5-53 yeast. Fab expression was induced using SGDCAA at 20 °C, 225 rpm for 36 h for subsequent sorting.

### 2.5. Cell-Based Biopanning

Mammalian cells were seeded at 4 × 10^5^ cells/well in 6-well plates (Corning 3516) and incubated at 37.0 °C with 5.0% CO_2_ for 2 days. After fixing the mammalian cells with 10% neutral buffered formalin, mammalian cells and the induced yeast library were washed with ice-cold DPBS containing calcium and magnesium supplemented with 0.1% BSA (wash buffer). The yeast mixture was added dropwise onto the mammalian cells. A cell density of 2.5 × 10^7^ yeast/cm^2^ in two wells was used for the first round of biopanning. The panning density was reduced in subsequent rounds of biopanning as the diversity of the library would decrease after biopanning. The density was decreased to 5 × 10^6^ yeast/cm^2^ in one well for the second round of biopanning and 5 × 10^6^ yeast/cm^2^ in one well for subsequent rounds (Table 1). The yeast cells were incubated with the mammalian monolayer at 4 °C for 2 h with gentle shaking. After the incubation, the supernatant containing non-binders was removed, and 1 mL/well of ice-cold wash buffer was added. The plates were further washed by rocking the plate 25 times and rotating the plate five times. This washing process was repeated another two times, followed by a final wash by rotating the plate ten times [14]. Brightfield images of the plate were acquired on an Olympus IX70 microscope (Melville, NY, USA) attached to a Nikon D5300 camera (Melville, NY, USA) controlled using Control My Nikon v.5.3 software. The microscope was also used to confirm the expression of AQP4 on the HEK293 cells through a mCherry fluorescence filter.

For positive selection biopanning, the non-binders were discarded, and 1 mL/well of ice-cold wash buffer was added after the washing steps. The cells on the plate were scraped off using a cell scraper the cells were collected. For negative selection biopanning, the non-binders were collected, and cells remaining on the plate were discarded.

Collected cells were then resuspended in 10 mL of low pH SDCAA, and a small fraction of the recovered cells were plated on SDCAA agar plates (Teknova S0543, Hollister, CA, USA) to quantify the number of yeast recovered after each round of biopanning. The remaining cells in the 10 mL of low pH SDCAA were expanded overnight at 30 °C, 225 rpm, followed by induction in SGDCAA for the subsequent round of biopanning.

### 2.6. Flow Cytometry and Fluorescence-Activated Cell Sorting (FACS)

In order to enrich for Fab-expressing clones, yeast were stained (without prior incubation with AQP4 antigen) with the anti-FLAG M2-FITC antibody (Sigma F4049) in staining buffer (1× DPBS with 0.5% BSA and 2 mM EDTA, pH 7.4) and yeast that expressed Fab (i.e., stained positive for the FLAG-tag) were sorted and collected into low pH SDCAA using a BD FACSAria Fusion cytometer. The gating strategy used is shown in Figure S2, which was drawn based on the wildtype AWY101 control.

For flow cytometry binding and screening, the monoclonal yeast or induced yeast library was washed with ice-cold staining buffer and incubated with 25 nM of 6xHis-tagged M23 or M23ex solubilized antigen at 4 °C for 30 min with gentle agitation on a platform shaker. Cells were washed three times and stained with 1:50 diluted anti-FLAG M2-FITC antibody (Sigma F4049) and APC anti-His Tag Antibody (Biolegend 362605, SanDiego, CA, USA) in staining buffer at 4 °C for 30 min with gentle agitation on a platform shaker. After washing the cells, clones that expressed Fab and showed binding to the antigen (i.e., stained positive for FLAG-tag and His-tag) were sorted and collected into low pH SDCAA using a BD FACSAria Fusion cytometer. Binding quadrants on the FITC vs. APC plots were drawn based on the 2B4 and IC05-2-2 negative binders. In the first round of sorting, yeast were collected under the yield collection mode to avoid depleting rare positive binders, but subsequent rounds of sorts were collected under purity mode to minimize the collection of non-binders. A higher percentage of recovered cells was observed after the first round compared to the second round of enrichment, which is likely due to the differences in collection modes (Table 2). Flow cytometry data analysis was conducted using FlowJo v10 (Ashland, OR, USA).

### 2.7. NGS Analysis of Sorted Library Composition

After each round of sorting, an aliquot of the expanded culture was used for high-efficiency yeast plasmid DNA extraction, as previously reported [20]. The KAPA HiFi HotStart ReadyMix (Roche 07958935001, Cape Town, South Africa) and primers (2YDrec_heavy_Vfor_MSrev1: TCTCGTGGGCTCGGAGATGTGTATAAGAGACAGNNNNCTGTTATTGCTAGCGTTTTAGCA, and 2YDrec_huIgH_Crev_MSfor1: TCGTCGGCAGCGTCAGATGTGTATAAGAGACAGNNNNAAGGCGCGCCTGTACTTGC) were used to amplify the VH genes from each library. The amplified VH genes then underwent PCR using barcoded primers to incorporate unique identifiers for each sample. Samples were sequenced on the Illumina 2 × 300 MiSeq platform. The raw FASTQ data were processed as previously described [21,22,23]. Briefly, the sequences were quality filtered, followed by V(D)J gene identification and annotation of CDR3 regions using IgBLAST [24]. Antibody clones were tracked across yeast sort rounds by CDR-H3 amino acid sequence, and their clonal enrichment ratios were calculated [22]. Enrichment ratios are based on the number of reads for each CDR-H3 amino acid sequence. The number of reads is affected by factors such as sequence length, GC-content, and experimental error in measuring yeast cell concentrations when mixing cell lines. Because the starting libraries were precisely quantified by NGS, shifts in enrichment ratios between selection rounds provide quantitative insight into enrichment. For biopanning, the enrichment ratios were calculated by comparing sequence prevalence in each round of biopanning to the pre-sorted (i.e., Round 0) library. For FACS, the enrichment ratios were calculated by comparing the sequence prevalence in each round of sorting to the initial unsorted Fab-expressing antibody library (i.e., Round 1 of V_L_+ sorting).

## 3. Results

### 3.1. Construction of Synthetic Yeast Screening Libraries to Model Anti-AQP4 Antibody Screening

Genomic sequences that encoded the V_H_ and V_L_ regions of the control and NMO-IgG antibodies were cloned into a pCT-VHVL-K1 yeast display vector backbone to generate the Fab surface display plasmids (Figure 1A) [19]. This platform utilizes the expression of the antigen-binding fragment (Fab) region of the antibody, which is fused with the a-agglutinin adhesion subunit (Aga2) protein. The antibody fragment-Aga2 fusion protein is captured by the a-agglutinin anchorage subunit (Aga1) protein anchored onto the cell wall of AWY101 yeast, allowing the antibody fragments to be displayed on the cell surface. The proper expression of the antibody fragment can be monitored through the FLAG expression tag.

The resulting plasmids were sequenced to confirm successful antibody cloning. After transforming the plasmids into yeast, proper Fab expression on the yeast was confirmed by probing for the FLAG-tag, and the monoclonal yeast was tested for their ability to bind to soluble M23 or M23ex AQP4 antigen by flow cytometry (Figure S3). Generally, yeast clones that expressed anti-AQP4 Fabs showed a weak binding signal in flow cytometry that was difficult to distinguish from yeast expressing control Fabs. All of the yeast clones (both AQP4 binders and non-binders) had <1% binding to the M23 antigen. Binding to the M23ex antigen was slightly more apparent for certain AQP4 binders, where 3.12% of the 10-1-121 Fab-expressing yeast were binding to the M23ex antigen compared to the 0.77% and 0.73% binding for the 2B4 and IC05-2-2 negative controls, respectively. In order to generate a synthetic yeast library to test different sorting strategies, monoclonal yeast cells were mixed with 49% of each non-binders (i.e., 2B4 and IC05-2-2 yeast) and 0.5% of each AQP4 binders (10-1-121, 10-1-153, 7-5-186, and 7-5-53 yeast) so that the majority of the synthetic yeast library contained non-AQP4 binding antibodies.

### 3.2. Monoclonal Anti-AQP4 NMO Antibodies Bind to M23-AQP4 Antigens

Prior to our library screening analyses, we first confirmed the ability of NMO-IgGs to bind to AQP4. M23-AQP4-expressing U87MG cells were incubated with control recombinant antibodies (2B4 or IC05-2-2) or recombinant monoclonal NMO-IgGs (10-1-121, 10-1-153, 7-5-186, or 7-5-53) for 2 h (Figure S4) [16,17,18]. No staining was observed with isotype controls, whereas staining patterns of the recombinant monoclonal NMO-IgGs overlapped with regions of AQP4 expression, confirming that these recombinant monoclonal NMO-IgGs exhibited binding specificity towards M23-AQP4.

### 3.3. AQP4-Expressing HEK293 as a Target for Cell-Based Biopanning

We used U87MG cells that stably express M23-AQP4 to validate our panel of soluble recombinant antibodies; however, several potential limitations exist for these cells in biopanning applications. First, only a fraction of M23-AQP4-expressing U87MG cells stained positive for AQP4, suggesting that they have limited expression of AQP4 (Figure S4). Although the reason for limited AQP4-expression is unclear, M23-AQP4 has been reported to induce apoptosis in U87MG cells [25]. Second, testing for AQP4 expression required the permeabilization and subsequent staining with a C-terminal specific rabbit anti-AQP4 antibody. To overcome these limitations, we used HEK293 cells that express M23-AQP4-mCherry or M23-AQP4ex-mCherry, which we found to have more consistent AQP4 expression and enabled direct AQP4 expression monitoring via the mCherry fluorescent signal (Figure S5).

Immunofluorescent staining of the AQP4-expressing HEK293 cells using recombinant antibodies showed recombinant monoclonal NMO-IgGs bound to the AQP4 expressed on the HEK293 cells. Staining with two hours of primary antibody incubation was effective, but overnight incubation resulted in non-specific cell staining for some clones (Figures S6 and S7).

### 3.4. Cell-Based Biopanning for Anti-AQP4 Antibody Identification

Our cell-based biopanning strategy consisted of three rounds of positive selection, then two rounds of negative selection, followed by two rounds of positive selection (seven rounds in total). In positive selection biopanning, the yeast library was incubated with mammalian cells that express AQP4 antigen for two hours at 4 °C with gentle shaking and binders were collected by cell scraping. Cell scraping would collect both the bound yeast cells and mammalian cells, but the mammalian cells did not grow under yeast culture conditions. During negative selection biopanning, the yeast library was incubated on mammalian cells that do not express AQP4 antigens under the same conditions, and the non-binders were collected by pipetting the supernatant (i.e., yeast cells in suspension) (Figure 1B). Our cell-based biopanning strategy was employed using M23-expressing U87MG cells, M23-expressing HEK293 cells, and M23ex-expressing HEK293 cells. The number of yeast cells recovered was not assessed after the first round of selection to avoid the loss of the rare positive binders. There was a higher percentage of yeast recovery during the initial three rounds of positive selection (7-fold [from 0.019% to 0.14%], 20-fold [from 0.0035% to 0.070%], and 22-fold increase [from 0.0028% to 0.063%] for M23 U87MG, M23 HEK293, and M23ex HEK293, respectively from Round 2 to Round 3) (Table 1). The first round of negative selection to remove non-specific binders resulted in the depletion of ~50% of the yeast population. However, few yeast were depleted after the subsequent negative selection (i.e., higher percent recovery), suggesting that negative selection was effective in removing many non-specific clones from the library.

**Figure 2:**
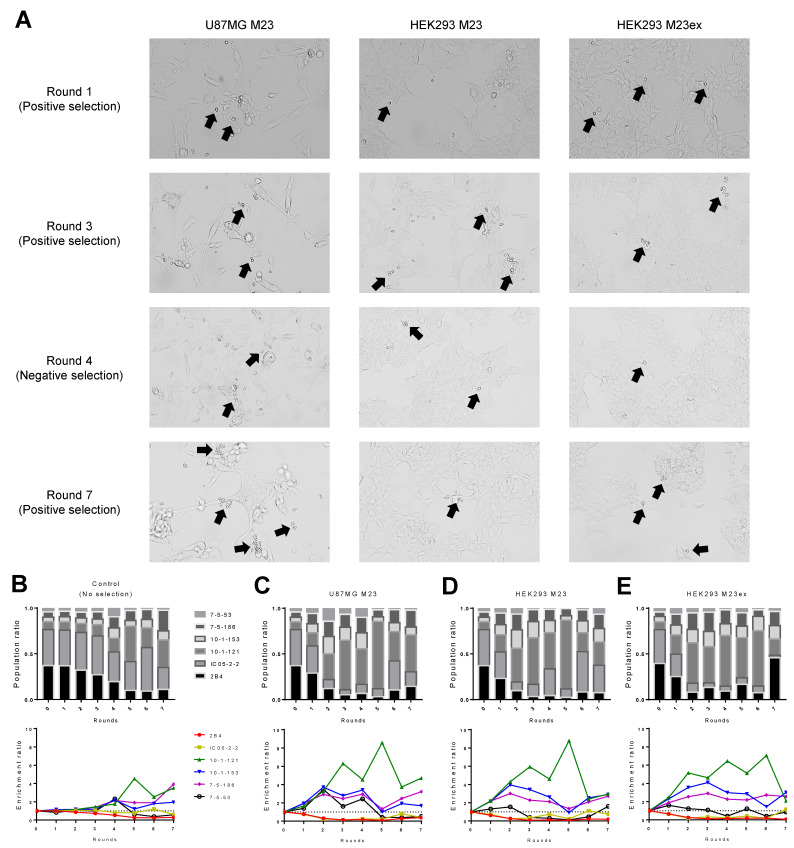
Cell-based biopanning results. (**A**) Representative brightfield images of biopanning after various rounds of selection. Black arrows show regions of yeast cells (small circular cells) binding to the adherent mammalian cells (large elongated cells). After each round of biopanning enrichment, NGS was performed to assess the population ratio and enrichment ratio for the (**B**) no selection control, and biopanning with (**C**) U87MG M23, (**D**) HEK293 M23, or (**E**) HEK293 M23ex adherent mammalian cells. For the no selection control, the yeast library was diluted without selection to monitor the natural drift of the population.

We analyzed the clonal composition of yeast libraries after sorting the yeast recovery to quantify the effectiveness of each functional screening method after each round of selection (Figure 2A and Figure S8). As a control, we also analyzed the yeast library passaged in the absence of functional selection to monitor general population drift. Consistent with our prior reports, the clonal composition of the yeast library was generally stable for up to three rounds of selection and demonstrated that the different yeast clones have fairly similar growth rates, although some drift was observed after three rounds (Figure 2B) [19]. Cell-based biopanning with M23-expressing U87MG cells resulted in the enrichment of the expected AQP4 binders after two rounds of enrichment, while non-AQP4 binders were depleted (Figure 2C). Biopanning was similarly effective for AQP4-expressing HEK293, although in this case, yeast expressing the 7-5-53 Fab did not become enriched (Figure 2D,E). Importantly, the enrichment of specific yeast clones occurred rapidly in sorted groups (beginning in Round 1 or 2) (Figure 2C–E), whereas natural population drift was minimal until Round 4 (Figure 2B).

### 3.5. FACS Strategy for Anti-AQP4 Clonal Enrichment

**Figure 3:**
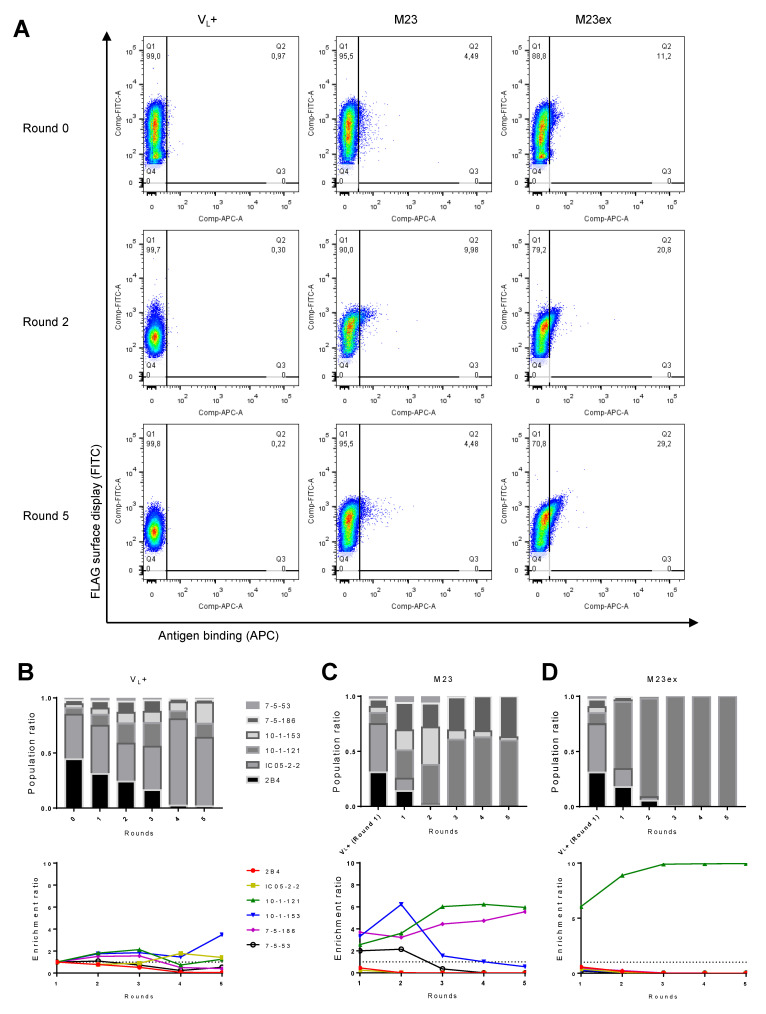
Soluble antigen FACS results. (**A**) FACS plot of sorting for V_L_+ control, and M23 and M23ex soluble antigen binders during Round 0 (i.e., pre-sort), Round 2, and Round 5. After each round of FACS enrichment, NGS was performed to assess the population ratio and enrichment ratio of the monoclonal yeast clones for the (**B**) V_L_+ control, and FACS enrichment with (**C**) M23, or (**D**) M23ex soluble antigen. For the V_L_+ control, the yeast library was selected for the yeast that displayed Fab determined by the expression of the FLAG tag.

We also sought to functionally screen our yeast library using FACS, where the library was incubated with fluorescent markers and solubilized antigens that allow concurrent detection of Fab surface display and AQP4 antigen binding. Yeast cells that properly express Fab were analyzed in one channel, and the yeast that both expressed Fab and also bound to the antigen were sorted and collected (Figure 1B, Table 2). We used NGS of sorted yeast populations to monitor library composition throughout successive rounds of functional screening (Figure 3A and Figure S9). We sorted the synthetic library for all clones that express Fab on their surface as a control (V_L_+) without any consideration of antigen binding. In the V_L_+ sorted library, the ratio of the clones did not remain constant (Figure 3B), and the population drift that we observed likely occurred due to differences in Fab expression between the different yeast clones. Nevertheless, during the first two rounds of sorting with the soluble M23 antigen, the expected AQP4 binders were enriched, and the non-AQP4 binders were depleted appropriately relative to the control V_L_+ population (Figure 3C). The differences in clonal composition observed between the control V_L_+ and antigen-sorted populations reflected the functional enrichment of the yeast clones that bind to the M23 antigen. After three rounds of enrichment, the prevalence of 10-1-153 and 7-5-53 AQP4-binding clones decreased, while 10-1-121 and 7-5-186 further enriched, suggesting clonal dominance can be substantial after >3 rounds of screening. When we repeated FACS screening with the soluble M23ex antigen, only the 10-1-121 AQP4-binding clone was enriched, with enrichment plateauing after three rounds of selection (Figure 3D). These data revealed that the M23 solubilized antigen was highly effective for functional screening of anti-AQP4 yeast, whereas the M23ex antigen showed more limited efficacy using known positive control clones.

## 4. Discussion

Here we validated the use of yeast-based antibody surface display for functional screening of anti-AQP4 membrane antigens, both via cell-based biopanning and by solubilized antigen FACS. Cell surface display technology is a powerful tool that can identify antibodies. Here we developed specialized methods to apply cell surface display against hydrophobic membrane proteins. Hydrophobic and membrane proteins such as AQP4 are very difficult to isolate and formulate for antibody discovery applications, making it challenging to discover antibodies against surface receptors and to understand autoimmune antibody responses.

Several surface display systems have been established for functional antibody screening, and there have been several reports describing antibody phage display against membrane protein targets [1,26,27]. Phage display offers larger library sizes compared to yeast [28,29]. However, yeast surface display is also popular in antibody discovery and engineering, as antibodies displayed by yeast can be closer to the native conformation based on its eukaryotic expression system, especially using a Fab expression format, and yeast display also shows less replication bias than phage display [12,19,29]. Mammalian surface display systems offer the advantage of producing full-length antibody displays with correct folding and post-translational processing but are limited by smaller library sizes and more complex techniques required to generate singly transduced cells encoding only a single antibody variant. Newer genetic engineering tools such as CRISPR/Cas9, transposase-based cloning platforms, and transcription activator-like effector nucleases (TALENs) offer the possibility of more efficient library generation, and thus mammalian display systems are now gathering momentum in the antibody discovery landscape [12,28].

Most previous anti-AQP4 antibody discoveries from human patients applied traditional single-cell sorting and sequencing of B cells, followed by antibody cloning, expression, and analysis for AQP4 antigen binding [6,30,31,32]. With a yeast display system, a library of antibodies can be screened directly *en masse* for antigen binding. Screening can be performed through methods such as cell-based biopanning and cell sorting (e.g., FACS or magnetic-activated cell sorting) [12,14]. Using a carefully designed synthetic yeast display library, we demonstrated both biopanning and FACS for antibody identification, using AQP4 as a model protein.

Cell-based biopanning is a rapid and cost-effective technique, as antibody staining and flow cytometry equipment are not needed for selection rounds. However, the mammalian cell lines require management, and there is also a high risk of non-specific binding to endogenous proteins on mammalian cells. To easily monitor the expression of AQP4, we used HEK293 cells that expressed the AQP4 membrane protein in a fusion format with an intracellular mCherry protein. We added the mCherry protein to the C-terminus of AQP4, as an N-terminal tag has previously been reported to disrupt OAP formation and recognition by NMO-IgGs [33]. To minimize non-specific binding and membrane internalization, we limited incubation time to 2 h, and incubation was performed under cold conditions (4 °C) [26]. Moreover, we employed negative selection screening to deplete non-specific binders from the library. Other groups have reported performing negative screening after a few rounds of positive screening [13] or performed negative screening prior to positive screening [1,26]. To prevent the loss of rare binders in the library, we performed positive selection before negative selection, but we believe that the appropriate steps can likely be achieved in any order, provided that both negative selection and positive selection are used appropriately.

We also implemented FACS screening as an anti-AQP4 selection tool, which allows gating for single cells and the selection for Fab-expressing clones. A disadvantage to FACS screening is that purified soluble antigen is required, which is especially challenging for hydrophobic membrane proteins such as AQP4 [34]. Detergents are sometimes used to help solubilize the protein, but they can occasionally disrupt the membrane protein at the same time. Moreover, membrane protein extraction and purification can sometimes make the protein unstable and compromise its native conformation. In this study, the AQP4 antigens were solubilized with the mild-detergent n-Dodecyl β-D-maltoside, which was shown to maintain their native protein conformation in a stable form [15]. Recent advances for antigen isolation and purification have also explored other solubilization methods (e.g., lipid nanodiscs) that allow membrane proteins to be presented in their native membrane-embedded environment, and we anticipate that these techniques will be increasingly used for antibody discovery [15,35].

Using our FACS-based approach, we found that sorting with the M23 antigen successfully selected for all AQP4-binding clones, but selection for 10-1-121 and 7-5-186 was slightly higher than 10-1-153. Since 10-1-153 and 7-5-53 antibodies have been reported to bind to extracellular loops C and E (i.e., Pattern 1), while 10-1-121 and 7-5-186 antibodies bind to extracellular loops A, C, and E of AQP4 (i.e., Pattern 2) [18,36], the M23 antigen may have preferential binding to Pattern 2 antibodies. Contrastingly, sorting with the M23ex antigen selected only the 10-1-121 clone successfully. This may suggest that the 10-1-121 antibody binds strongly to the extended C-terminus region. In contrast, similar clones were enriched between M23 and M23ex with cell-based biopanning. The difference that we observed in selection efficacy between biopanning and FACS is not surprising as the C-terminus of AQP4 exists intracellularly. Therefore, the extended domain would not be readily accessible in the cell-based antigen display format of biopanning but is freely accessible in the soluble antigen display format. Moreover, structural differences may exist when AQP4 is displayed on the cell membrane compared to when it is solubilized.

A recent study also investigated a biofloating screening technique [37]. In contrast to using antigen-expressing adherent cells in biopanning as we have performed here, the biofloating method uses suspended cells, enabling potential integration with FACS sorting. The biofloating method could thus potentially combine the advantages associated with cell sorting (e.g., quantitative analysis of binding interactions) with the advantages of cell-based antigen display. However, the authors noted that biofloating might not be effective in selecting weak antibody binders, which is an area where soluble antigen-based FACS excels [37,38,39].

Our synthetic library was designed to contain seven unique clones, whereas a native antibody library of the B-cell repertoire from an autoimmune patient could contain many more diverse variants. Our efforts with this more simple control library provided a solid foundation to evaluate the efficacy of different screening strategies and provide strong background data for subsequent screening studies of more complex patient libraries. We also focused on the M23 isoform of AQP4 for screening studies, as previous studies demonstrated that the NMO-IgGs better recognize the OAP-forming M23-AQP4 compared to M1-AQP4 [8,40]. Native AQP4 found in humans comprises a mixture of the different isoforms of AQP4, and we will use both the M23 and M1 isoforms for the interrogation of future autoimmune patient libraries.

Our study has validated the use of both cell-based biopanning and FACS to effectively select antibody clones that bind to the membrane protein, AQP4, using a yeast display format. We found that the cell-based biopanning technique was superior to other alternatives using a synthetically generated antibody library. These data agree with other recent reports of cell-based assays offering higher sensitivity and specificity in AQP4 autoantibody detection than AQP4 ELISA assays that use purified antigens [41]. Moreover, compared to FACS, sorting by cell-based biopanning is quicker, avoids the need for specialized equipment, and better preserves the native conformation of the antigen to facilitate a more effective selection of antigen-binding clones. FACS is still an important approach and is highly effective for understanding antibody responses against specific isoforms and gene variants that have been specifically purified, in contrast to cell-based display where the composition of the antigen is less certain. In addition, depleting non-specific binders via negative selection is critical for biopanning, especially when working with a more diverse and complex patient library.

In our hands, sorts performed best if kept in the first three rounds of sorting; consistent with previous reports, no further enrichment was observed beyond four rounds of sorting [42]. We used the AWY101 yeast strain for library generation, but the EBY100 yeast strain could also be used [13,43]. A strategy of two rounds of positive selection followed by one round of negative selection should be sufficient based on our data here, with the analysis of effective antibody clones performed by highly sensitive NGS. We look forward to applying these validated techniques in subsequent studies for the discovery of antibodies targeting AQP4 and against other relevant membrane protein targets. Future studies will apply our yeast display, biopanning, and FACS techniques to mine anti-NMO antibody responses and advance the understanding of disease and the development of new precision therapies for NMO.

## Figures and Tables

**Table 1:** Summary of cell-based biopanning parameters and enrichment.

Cell-Based Biopanning Round		1	2	3	4	5	6	7
Number of yeast applied		4.8 × 10^8^	4.8 × 10^7^	2.9 × 10^7^	2.9 × 10^7^	2.9 × 10^7^	2.9 × 10^7^	2.9 × 10^7^
Yeast density (yeast/cm^2^)		2.5 × 10^7^	5.0 × 10^6^	3.0 × 10^6^	3.0 × 10^6^	3.0 × 10^6^	3.0 × 10^6^	3.0 × 10^6^
Surface area (cm^2^)		19	9.5	9.5	9.5	9.5	9.5	9.5
Selection type		+	+	+	−	−	+	+
U87MG M23	Number of recovered yeast	N.D.	9.1 × 10^3^	3.9 × 10^4^	1.4 × 10^7^	3.3 × 10^7^	8.4 × 10^4^	9.0 × 10^3^
	Percent recovery (%)	N.D.	0.019	0.14	48	120 *	0.30	0.032
HEK293 M23	Number of recovered yeast	N.D.	1.7 × 10^3^	2.0 × 10^4^	1.3 × 10^7^	3.8 × 10^7^	9.5 × 10^3^	3.5 × 10^3^
	Percent recovery (%)	N.D.	0.0035	0.070	45	130 *	0.033	0.012
HEK293 M23ex	Number of recovered yeast	N.D.	1.4 × 10^3^	1.8 × 10^4^	1.4 × 10^7^	2.7 × 10^7^	1.3 × 10^4^	3.0 × 10^3^
	Percent recovery (%)	N.D.	0.0028	0.063	49	93	0.044	0.011

* Values larger than the theoretical maximum of 100%. This may reflect differences in the methods of counting yeast, where the number of yeast applied was estimated from OD readings (where 1 OD = 1 × 10^7^ yeast cells) and the number of yeast recovered was estimated from colony plate counting.

**Table 2:** Summary of FACS library screening parameters and enrichment.

Labeled Antigen FACS Round		1	2	3	4	5
Collection Mode		Yield	Purity	Purity	Purity	Purity
V_L_+	Number of yeast applied	5.0 × 10^6^	5.0 × 10^6^	5.0 × 10^6^	2.5 × 10^6^	2.5 × 10^6^
	Number of recovered yeast *	209,914	217,913	606,383	459,926	314,037
	Percent recovery (%)	4.2	4.4	12	18	13
M23	Number of yeast applied	5.0 × 10^7^	5.0 × 10^7^	5.0 × 10^7^	5.0 × 10^7^	5.0 × 10^7^
	Number of recovered yeast *	8693	1321	12,410	4047	1582
	Percent recovery (%)	0.017	0.0026	0.025	0.0081	0.0032
M23ex	Number of yeast applied	5.0 × 10^7^	5.0 × 10^7^	5.0 × 10^7^	5.0 × 10^7^	5.0 × 10^7^
	Number of recovered yeast *	24,049	7576	19,967	31,089	13,227
	Percent recovery (%)	0.048	0.015	0.040	0.062	0.026

* Number of yeast collected was assumed to be equal to the number of sorted event counts.

## Data Availability

All related data and methods are presented in this paper. Additional inquiries should be addressed to the corresponding author.

## Supplementary Materials

The following supporting information can be downloaded at: https://www.mdpi.com/article/10.3390/antib11020039/s1, Figure S1. Overview of the yeast display enrichment cycle. A DNA plasmid library is transformed into a yeast-surface display platform, generating the (1) yeast display library, which contains yeast that express and display the encoded antibody fragment protein on the cell surface. (2) Yeast library is exposed to target antigen (i.e., AQP4). (3) Desired AQP4 binders are selected and unbound yeast (i.e., non-AQP4 binders) are discarded. (4) The selected yeast are amplified, which can then be subject to steps 1–4 for further enrichment rounds. After enrichment, (5) plasmids are isolated from yeast cells are sequenced for analysis. Figure S2. Gating strategy to select for clones expressing Fab surface based on unmodified AWY101 yeast. The V500 channel was used as a dump channel. Figure S3. Monoclonal yeast binding to (A) M23 or (B) M23ex AQP4 soluble antigen. Figure S4. Anti-AQP4 recombinant antibodies (rAbs) bind to AQP4-expressing U87MG cells but not to wild-type U87MG cells. Representative images of immunofluorescent staining using 2B4 negative control recombinant anti-measles virus nucleocapsid protein antibodies, IC05-2-2 negative control recombinant antibodies derived from a meningitis patient, or anti-AQP4 recombinant antibodies 10-1-121, 10-1-153, 7-5-186, or 7-5-53. Staining was performed on M23-AQP4-expressing U87MG cells (left) or wildtype (non-M23-AQP4-expressing) U87MG cells (right). DAPI nuclear staining is in purple, recombinant antibody staining is in green, and AQP4 staining is in red. Figure S5. AQP4mCherry-expressing HEK293 cells allow for easy monitoring of AQP4 expression. Representative images of AQP4-expressing cell lines U87MG and HEK293. Hoechst 33342 nuclear stain is shown in blue, mCherry fluorescence is shown in red, and AQP4 stain (rabbit anti-AQP4 primary antibody and Alexa Fluor 647 goat anti-rabbit secondary antibody) is shown in magenta. Figure S6. Anti-AQP4 recombinant antibodies bind to AQP4-expressing HEK293 cells. (A) Representative immunofluorescence images of control (2B4 and IC05-2-2) and anti-AQP4 (10-1-121, 10-1-153, 7-5-186, and 7-5-53) antibodies binding to wildtype (WT) HEK293, HEK293 expressing M23-AQP4-mCherry, or HEK293 expressing M23-AQP4ex-mCherry. (B) Ratio of recombinant antibody binding signal relative to M23-AQP4 expression signal (i.e., mCherry signal). (C) Ratio of recombinant antibody binding signal relative to M23-AQP4ex expression signal (i.e., mCherry signal). Recombinant antibodies were incubated overnight. Figure S7. Non-specific binding to U87MG cells with increased incubation time. Representative images of 2B4 and IC05-2-2 control recombinant antibodies binding to M23-AQP4-expressing U87MG cells after (A) 2 h or (B) overnight incubation. Figure S8. Brightfield images of cell-based biopanning after each round of selection. Figure S9. FACS screening data after each round of selection. For non-antigen enrichment control, the initial yeast library was selected for yeast that express Fabs (i.e., V_L_+). The yeast library was subject to enrichment to soluble AQP4 M23 antigen or M23ex antigen.
