# Supplementary material for: Strategies to Screen Anti-AQP4 Antibodies from Yeast Surface Display Libraries
Source: Antibodies (Basel). 2022 Jun 5;11(2):39. doi: 10.3390/antib11020039 (PMC9220140; doi:10.3390/antib11020039)
Supplement: Supplementary file 1 [file antibodies-11-00039-s001.zip › antibodies-1720721-supplementary.pdf]

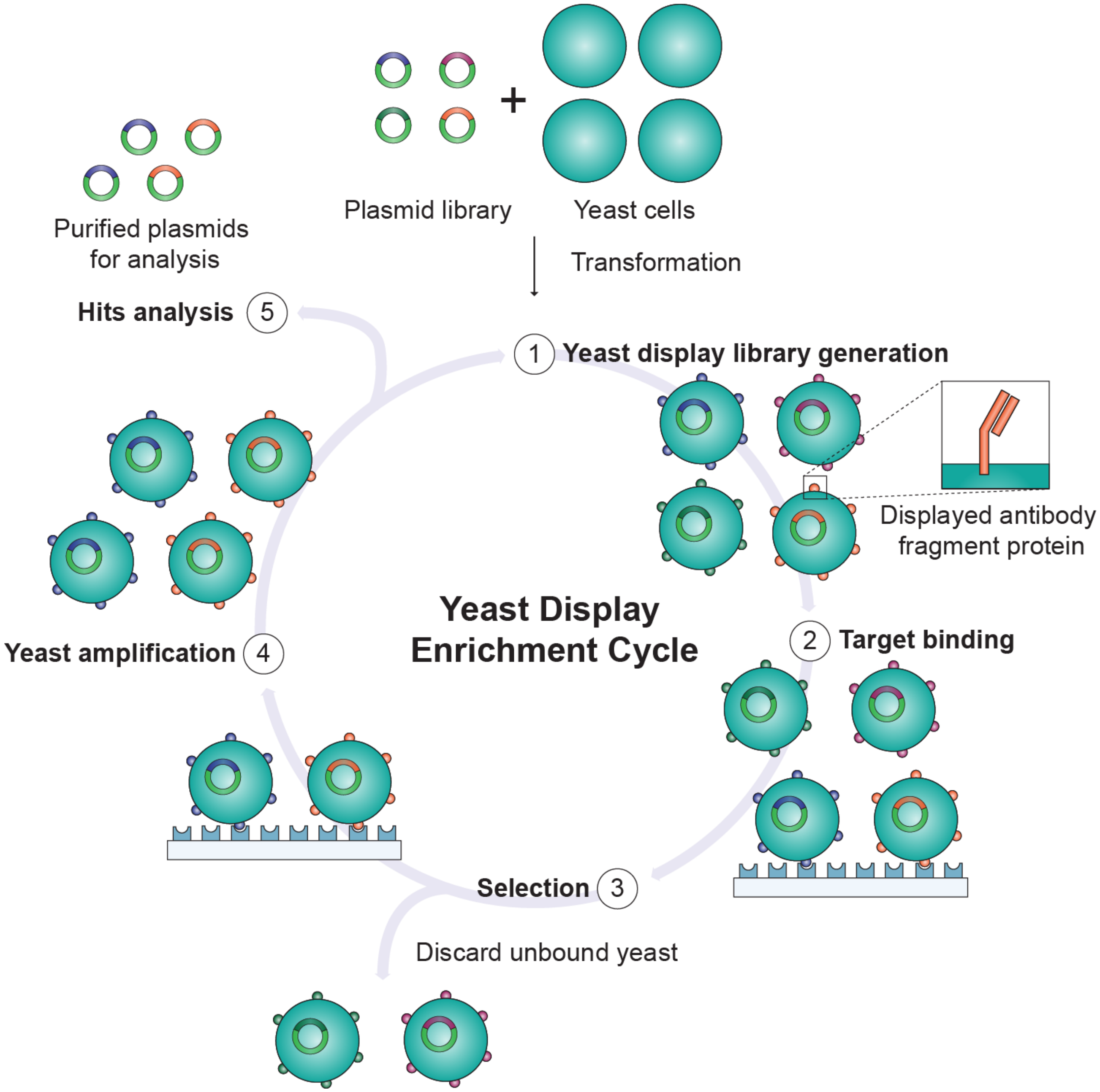

Figure S1. Overview of the yeast display enrichment cycle. A DNA plasmid library is transformed into a yeast-surface display platform, generating the (**1**) yeast display library, which contains yeast that express and display the encoded antibody fragment protein on the cell surface. (**2**) Yeast library is exposed to target antigen (i.e., AQP4). (**3**) Desired AQP4 binders are selected and unbound yeast (i.e., non-AQP4 binders) are discarded. (**4**) The selected yeast are amplified, which can then be subject to steps 1-4 for further enrichment rounds. After enrichment, (**5**) plasmids are isolated from yeast cells are sequenced for analysis.

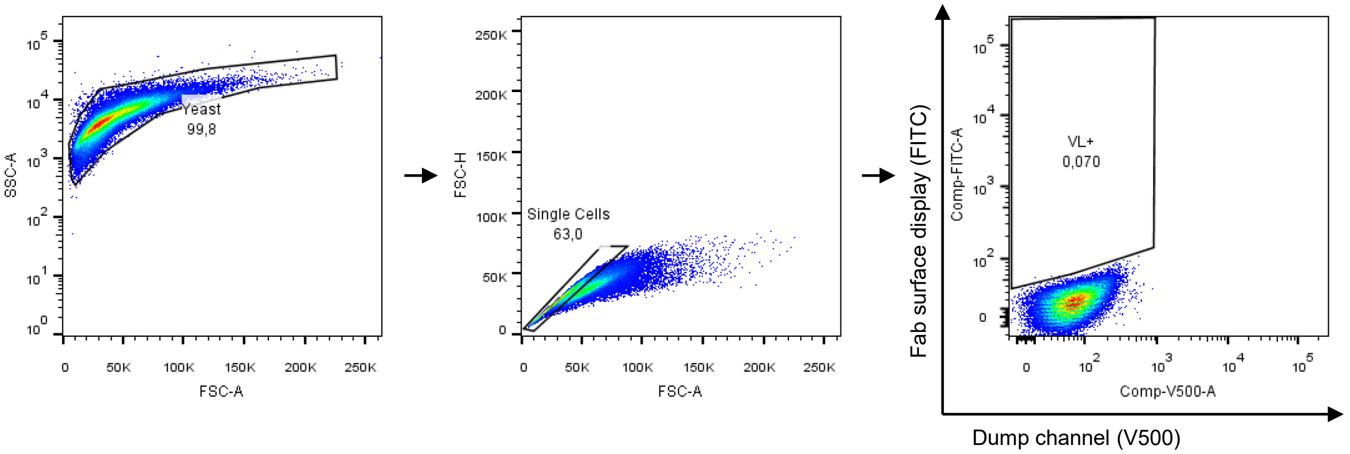

Figure S2. Gating strategy to select for clones expressing Fab surface based on unmodified AWY101 yeast. The V500 channel was used as a dump channel.

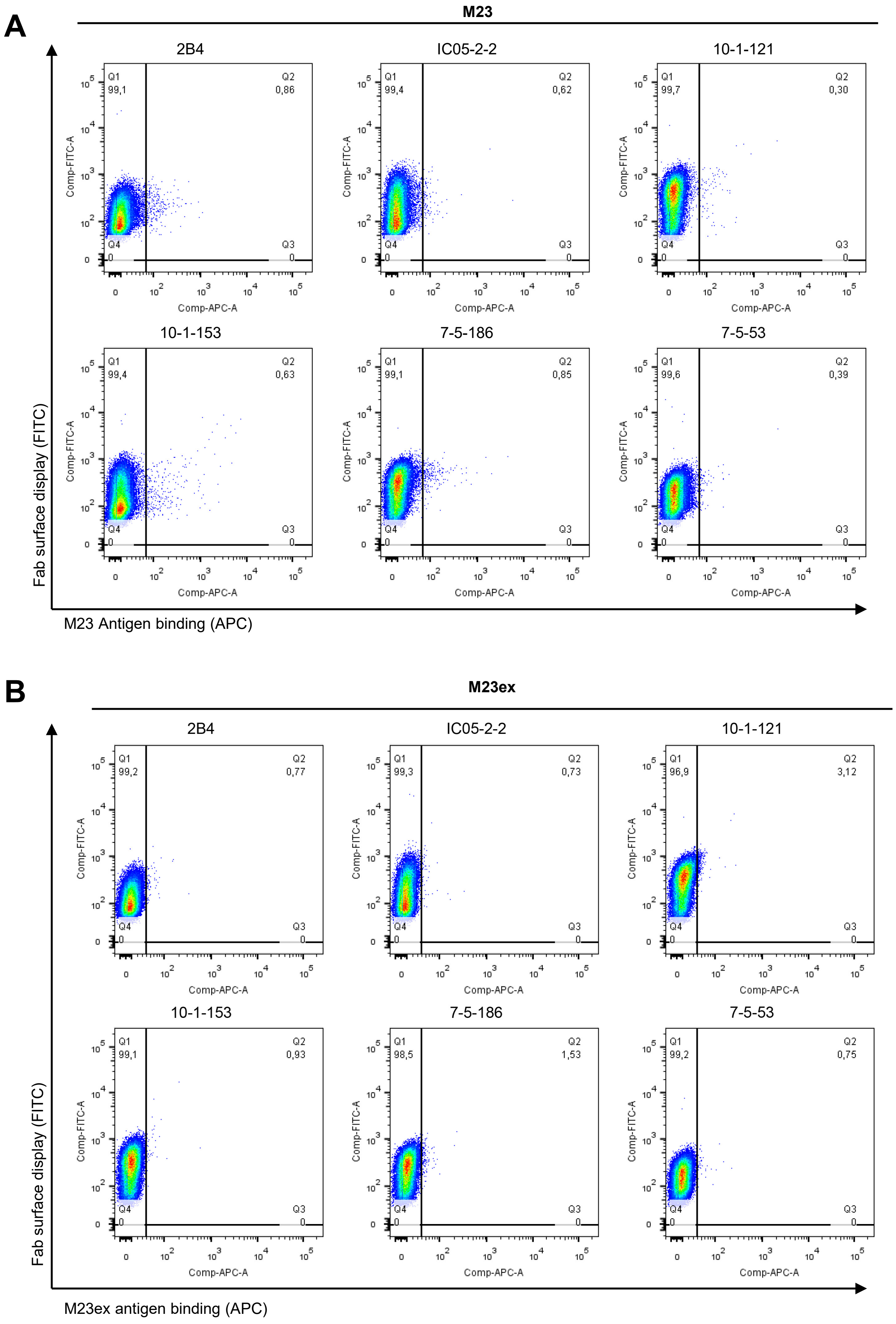

Figure S3. Monoclonal yeast binding to (**A**) M23 or (**B**) M23ex AQP4 soluble antigen.

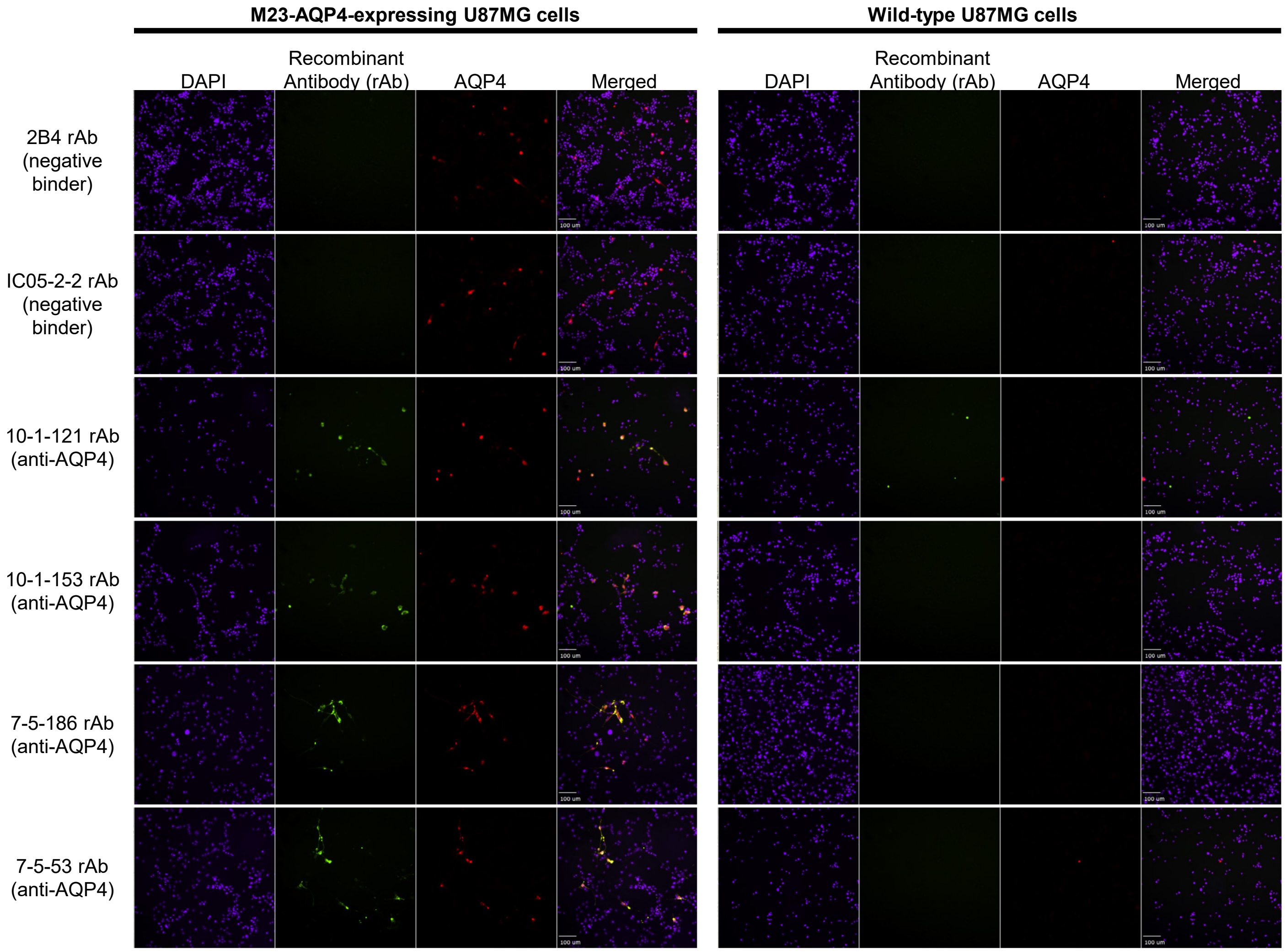

Figure S4. Anti-AQP4 recombinant antibodies (rAbs) bind to AQP4-expressing U87MG cells but not to wild-type U87MG cells. Representative images of immunofluorescent staining using 2B4 negative control recombinant anti-measles virus nucleocapsid protein antibodies, IC05-2-2 negative control recombinant antibodies derived from a meningitis patient, or anti-AQP4 recombinant antibodies 10-1-121, 10-1-153, 7-5-186, or 7-5-53. Staining was performed on M23-AQP4-expressing U87MG cells (left) or wildtype (non-M23-AQP4-expressing) U87MG cells (right). DAPI nuclear staining is in purple, recombinant antibody staining is in green, and AQP4 staining is in red.

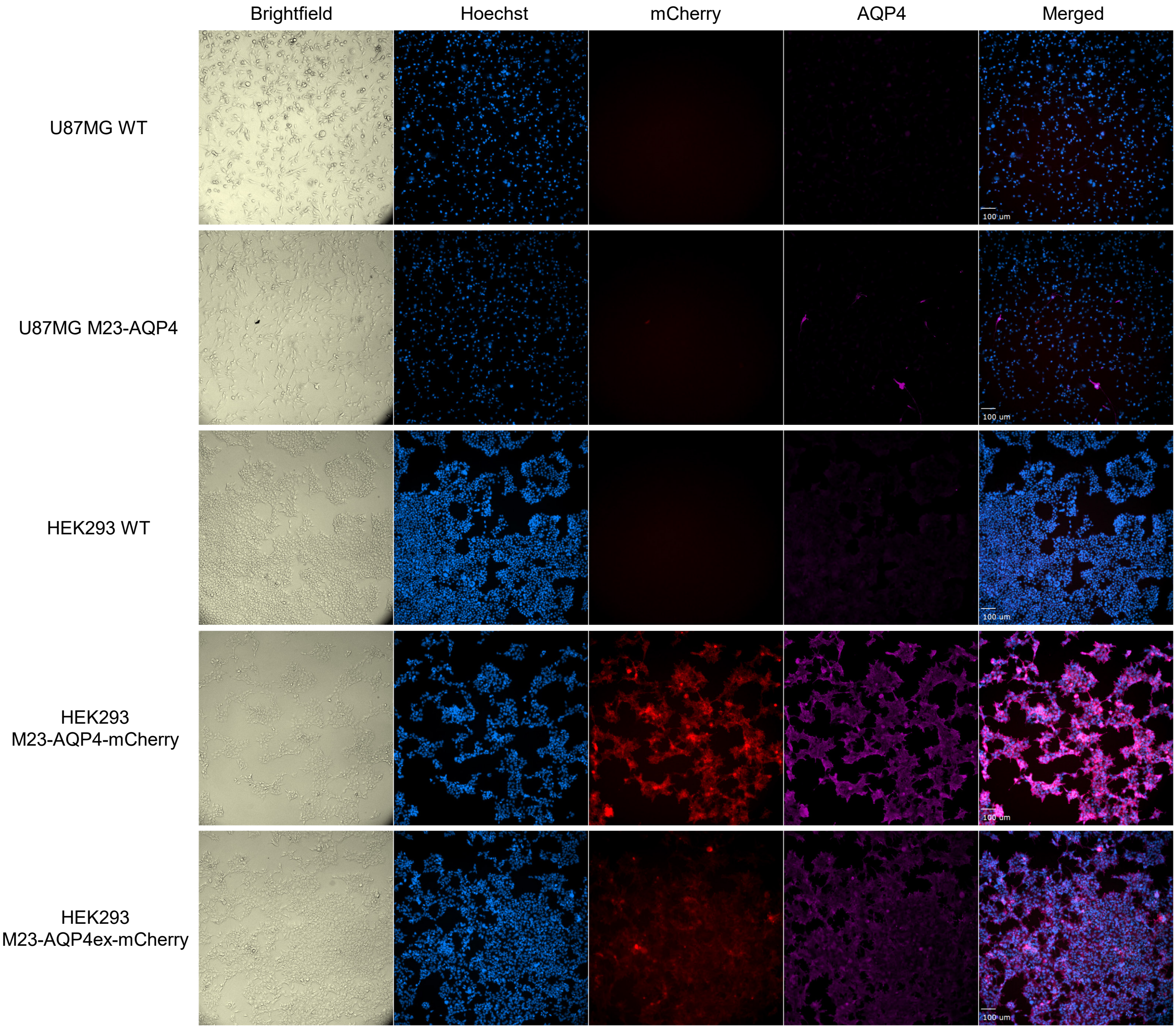

Figure S5. AQP4mCherry-expressing HEK293 cells allow for easy monitoring of AQP4 expression. Representative images of AQP4-expressing cell lines U87MG and HEK293. Hoechst 33342 nuclear stain is shown in blue, mCherry fluorescence is shown in red, and AQP4 stain (rabbit anti-AQP4 primary antibody and Alexa Fluor 647 goat anti-rabbit secondary antibody) is shown in magenta.

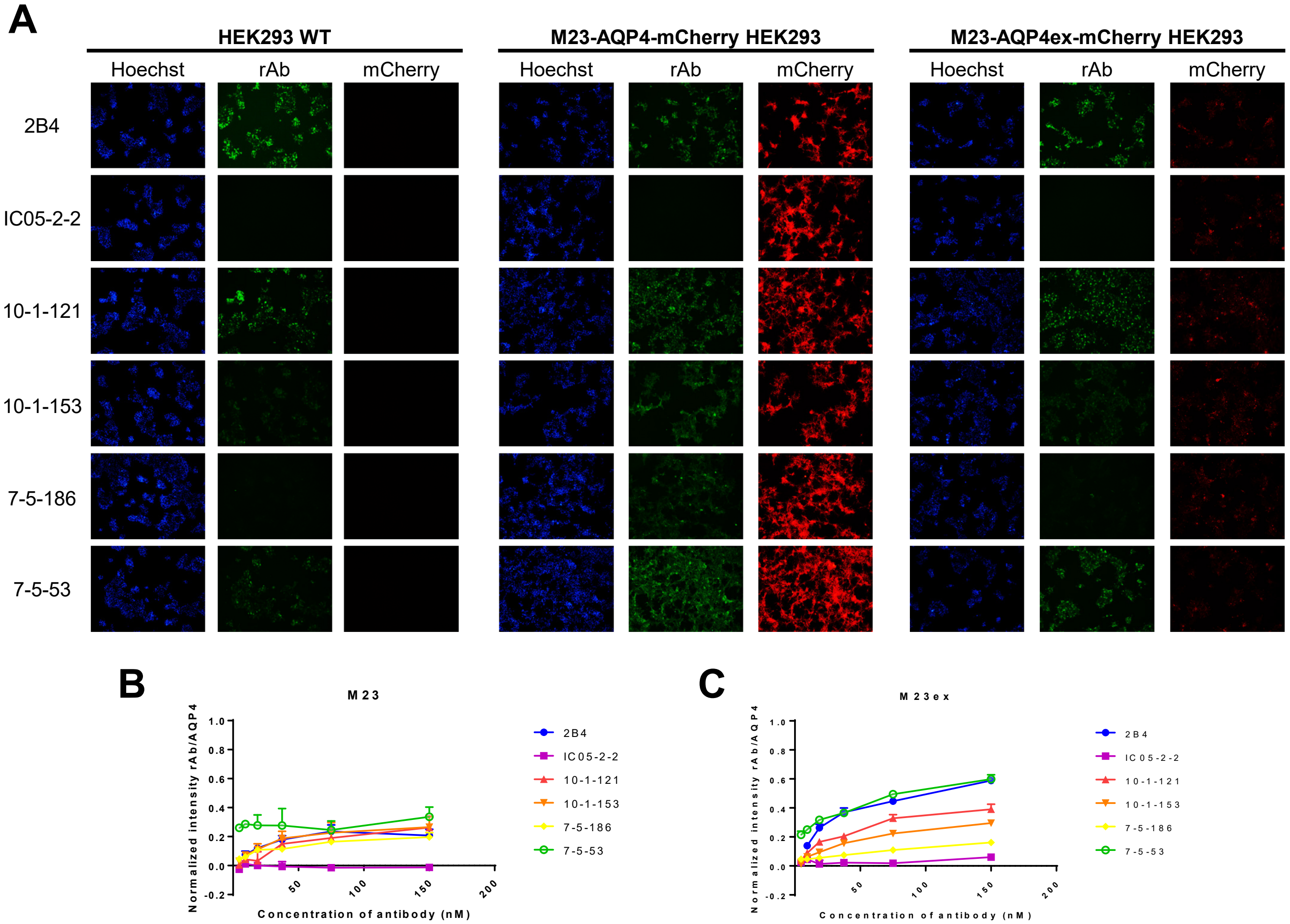

Figure S6. Anti-AQP4 recombinant antibodies bind to AQP4-expressing HEK293 cells. (**A**) Representative immunofluorescence images of control (2B4 and IC05-2-2) and anti-AQP4 (10-1-121, 10-1-153, 7-5-186, and 7-5-53) antibodies binding to wildtype (WT) HEK293, HEK293 expressing M23-AQP4-mCherry, or HEK293 expressing M23-AQP4ex-mCherry. (**B**) Ratio of recombinant antibody binding signal relative to M23-AQP4 expression signal (i.e., mCherry signal). (**C**) Ratio of recombinant antibody binding signal relative to M23-AQP4ex expression signal (i.e., mCherry signal). Recombinant antibodies were incubated overnight.

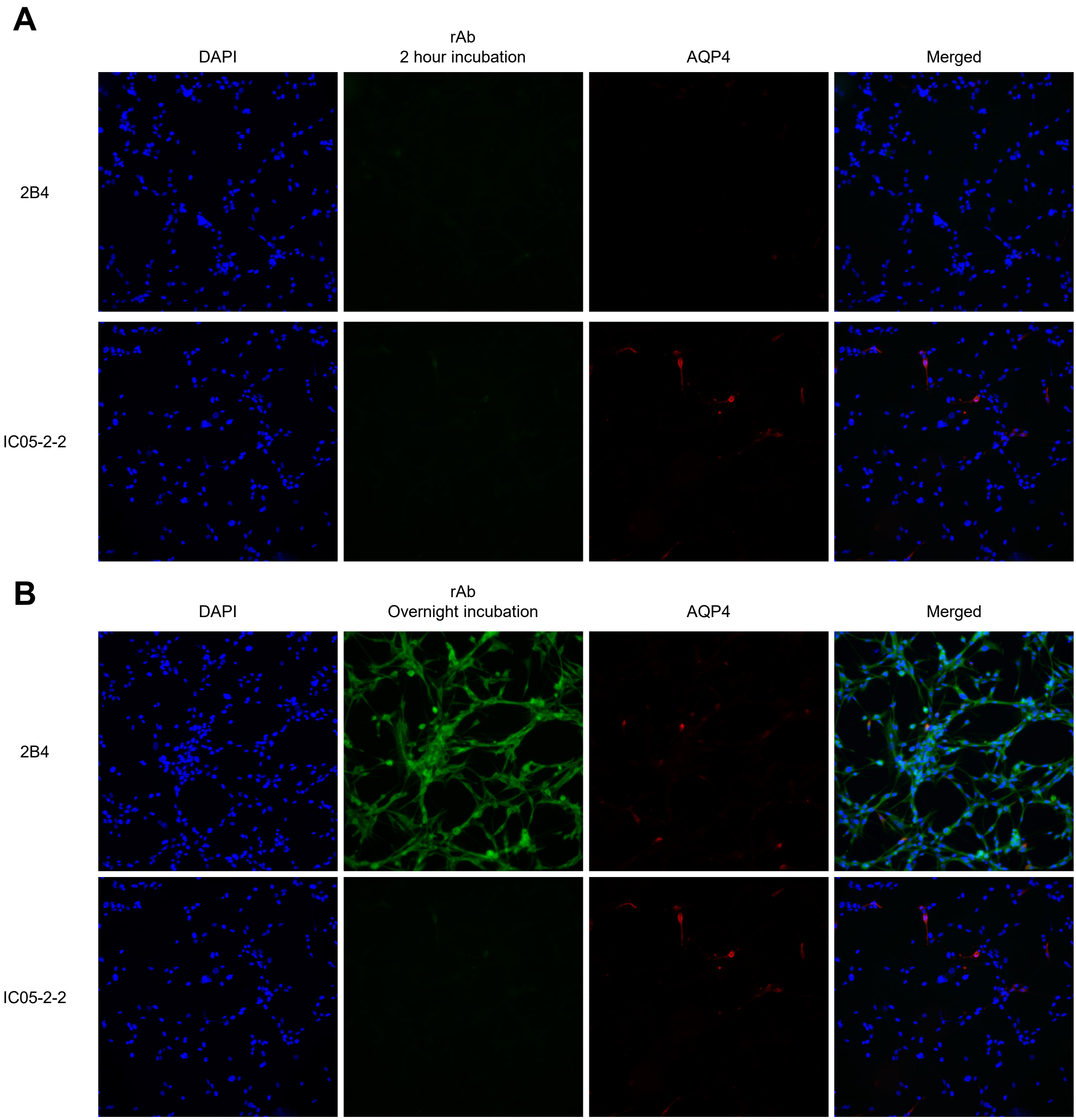

Figure S7. Non-specific binding to U87MG cells with increased incubation time. Representative images of 2B4 and IC05-2-2 control recombinant antibodies binding to M23-AQP4-expressing U87MG cells after (**A**) 2 h or (**B**) overnight incubation.

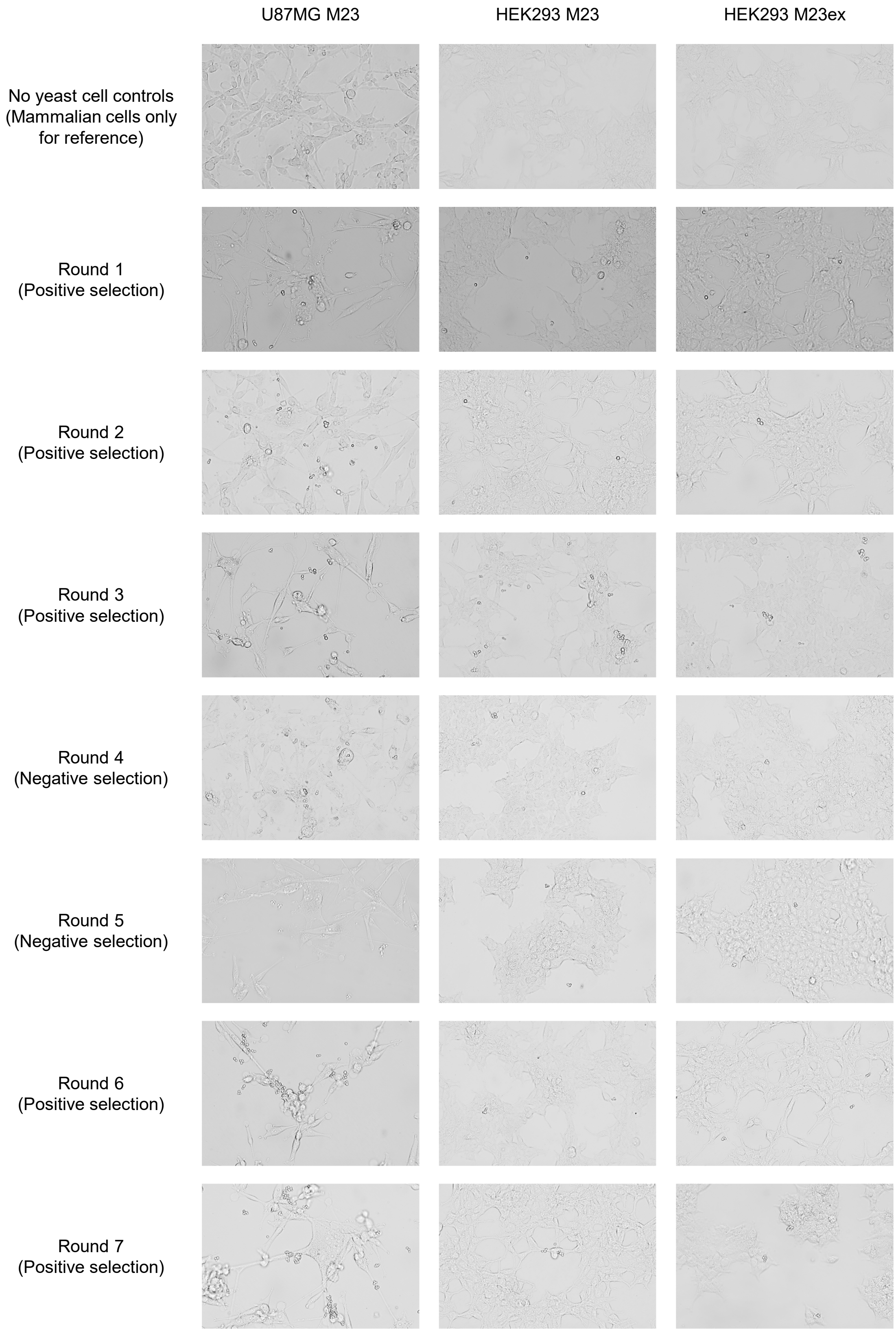

Figure S8. Brightfield images of cell-based biopanning after each round of selection.

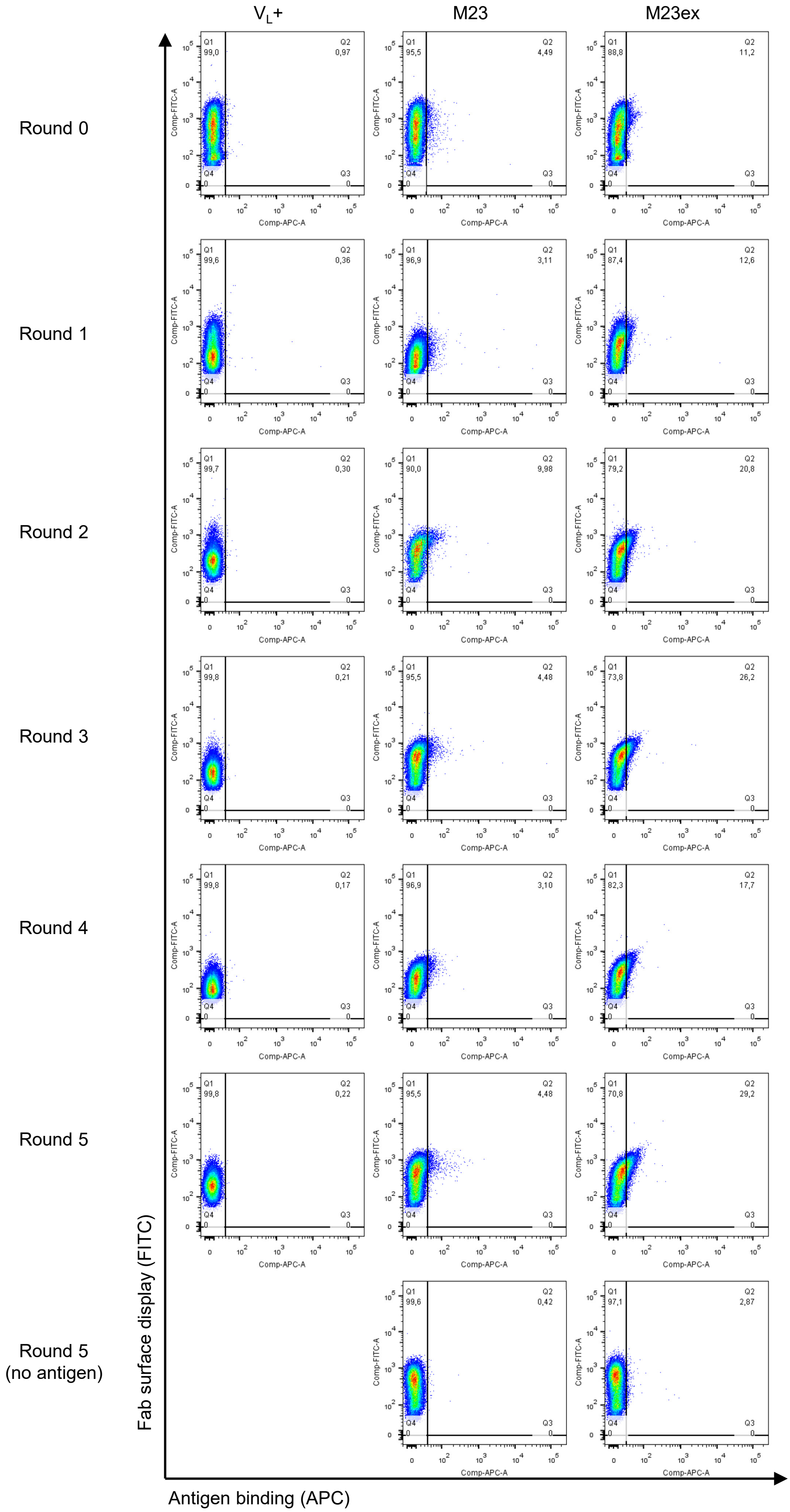

Figure S9. FACS screening data after each round of selection. For non-antigen enrichment control, the initial yeast library was selected for yeast that express Fabs (i.e., V_L+). The yeast library was subject to enrichment to soluble AQP4 M23 antigen or M23ex antigen.
